# Supplementary material for: In Vitro Modulation of Spontaneous Activity in Embryonic Cardiomyocytes Cultured on Poly(vinyl alcohol)/Bioglass Type 58S Electrospun Scaffolds
Source: Nanomaterials (Basel). 2024 Feb 17;14(4):372. doi: 10.3390/nano14040372 (PMC10892114; doi:10.3390/nano14040372)
Supplement: Supplementary file 1 [file nanomaterials-14-00372-s001.zip › Supplementary Material.pdf]

Supplementary Material

In Vitro Modulation of Spontaneous Activity in Embryonic Cardiomyocytes Cultured on Poly (Vinyl Alcohol)/Bioglass Type 58S Electrospun Scaffolds

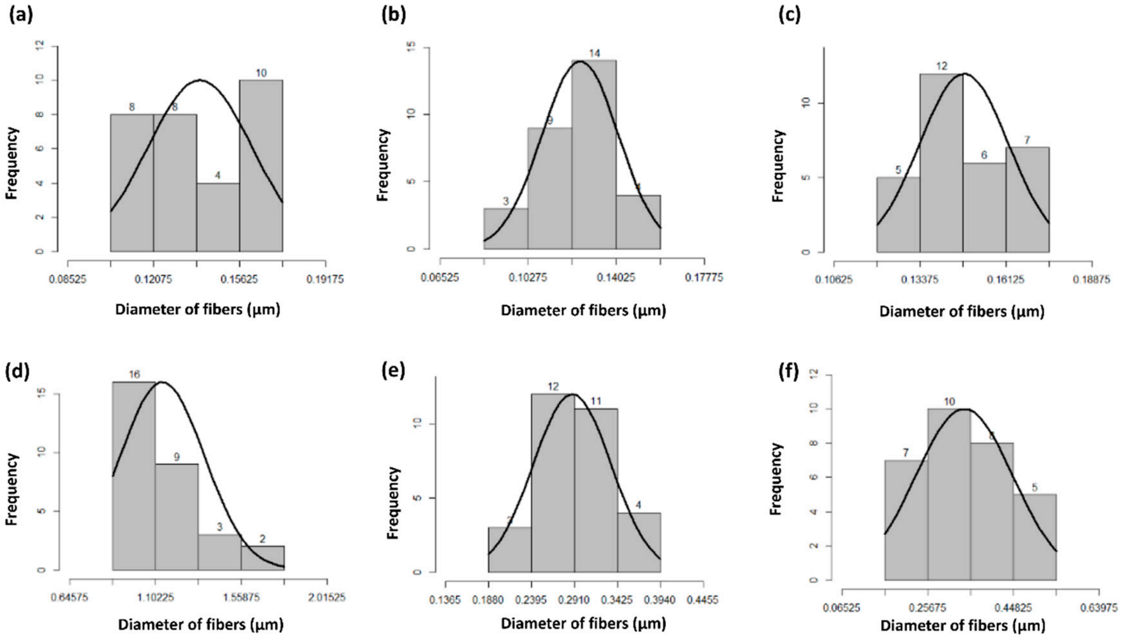

Figure S1. Histograms of fiber diameter PVA scaffolds with different Bg concentrations before cross-linking. (a) 5% hybrid, (b) 10% hybrid, (c) 15% hybrid; (d) 20% hybrid; (e) 25% hybrid, and (f) 30% hybrid.

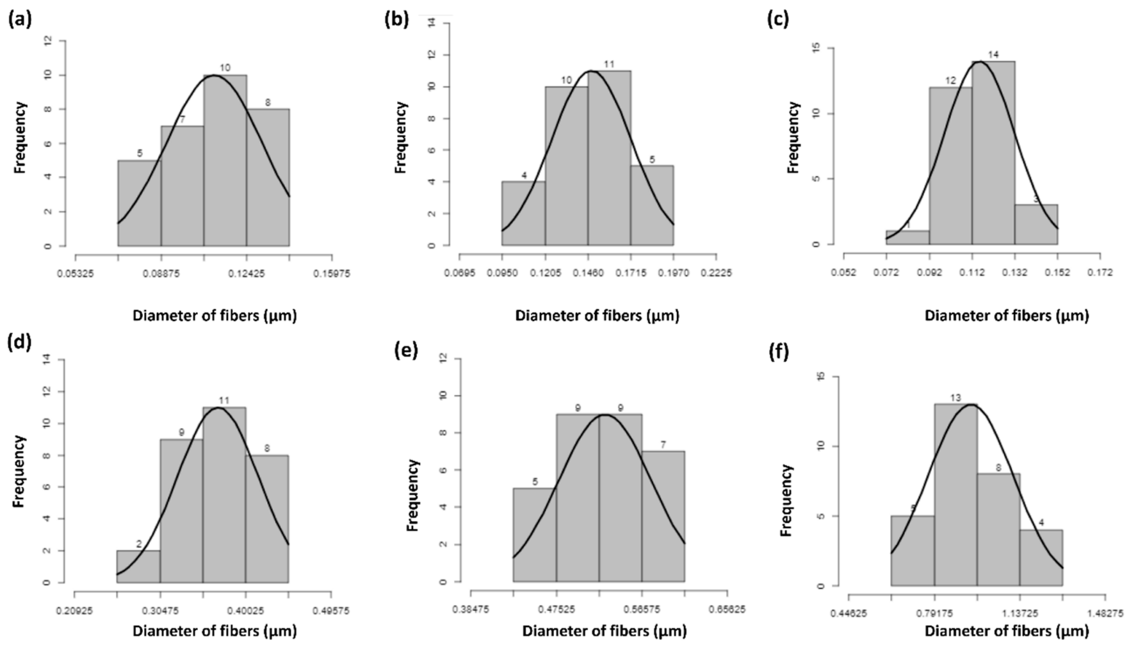

Figure S2. Histograms of fiber diameter PVA scaffolds with different Bg concentrations after cross-linking. (a) 5% hybrid, (b) 10% hybrid, (c) 15% hybrid; (d) 20% hybrid; (e) 25% hybrid, and (f) 30% hybrid.

Table S1. Functional groups observed by ATR-FTIR in PVA/Bg hybrid scaffolds.

**Bioglass content**	**Representative signals of the functional groups (cm^{-1})**										
	O-H	-OH	$-CH_2$	H_2C-CO	HC-OH	C-O	C-C	Si-O-Si (as)	Si-O-Si (s)	Si-O-Si (δ)	Si-O-Ca
**0 %**	3300	-	2940, 2909	1420	1327	1087	840	---	---	---	---
**5 %**	3300	-	2945, 2916	1422	1327	1084	840	--	---	---	--
**10 %**	3300	-	2945, 2916	1422	1327	1084	840	----	---	---	----
**15 %**	3300	-	2945, 2916	1422	1327	1084	840	----	---	---	----
**20 %**	3300	1659	2945, 2916	1422	1326	1089	832	1037	930	832	957
**25 %**	3300	1659	2942, 2908	1416	1327	1085	845	1042	920	830	958
**30 %**	3227	1655	2938, 2913	1416	1327	1080	825	1031	930	825	955
**Bg**	3493, 3443	1628, 1612	----	----	----	----	---	1042	932	789	956

(as), refers to asymmetric vibration, (s) symmetric vibration, and (δ) bending vibration.

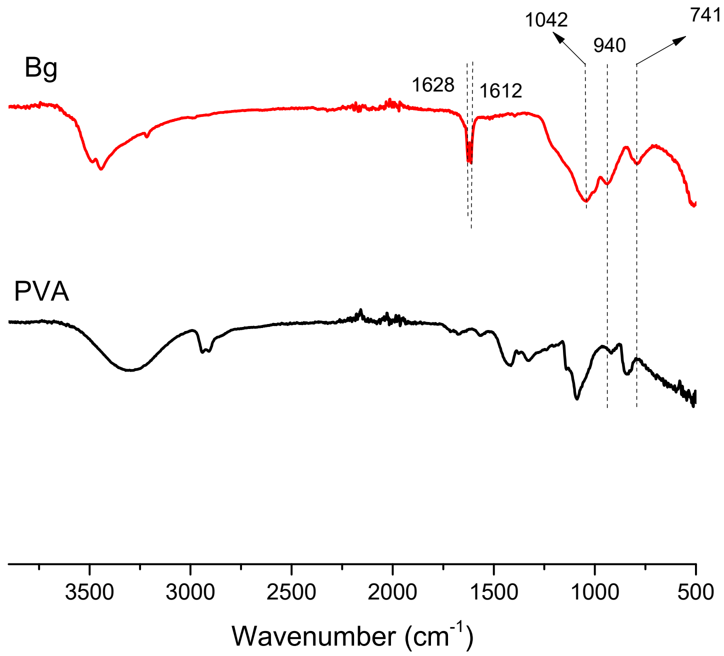

Figure S3. FTIR-ATR spectrum of PVA and 58S bioglass.

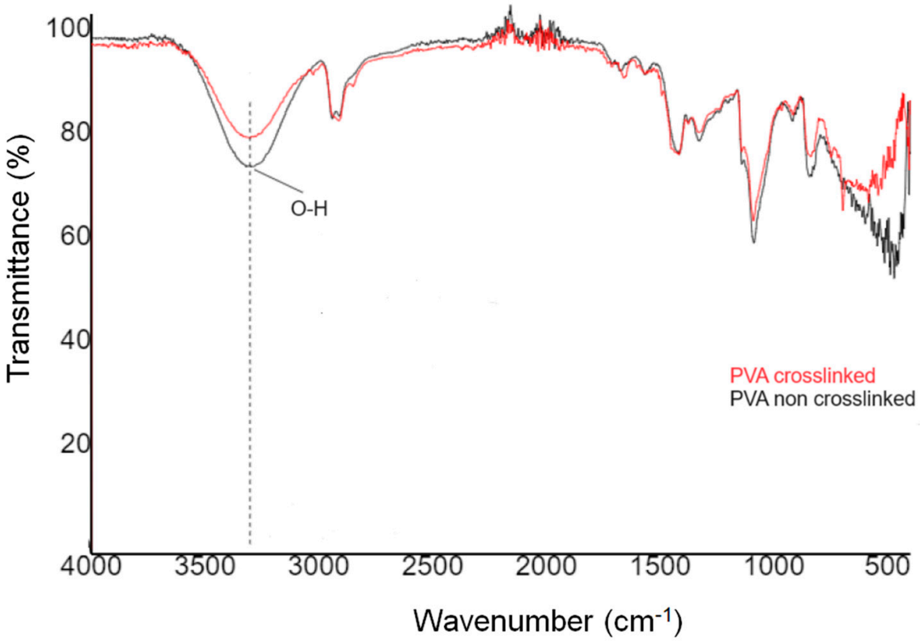

Figure S4. FTIR-spectra of PVA before and after crosslinking.

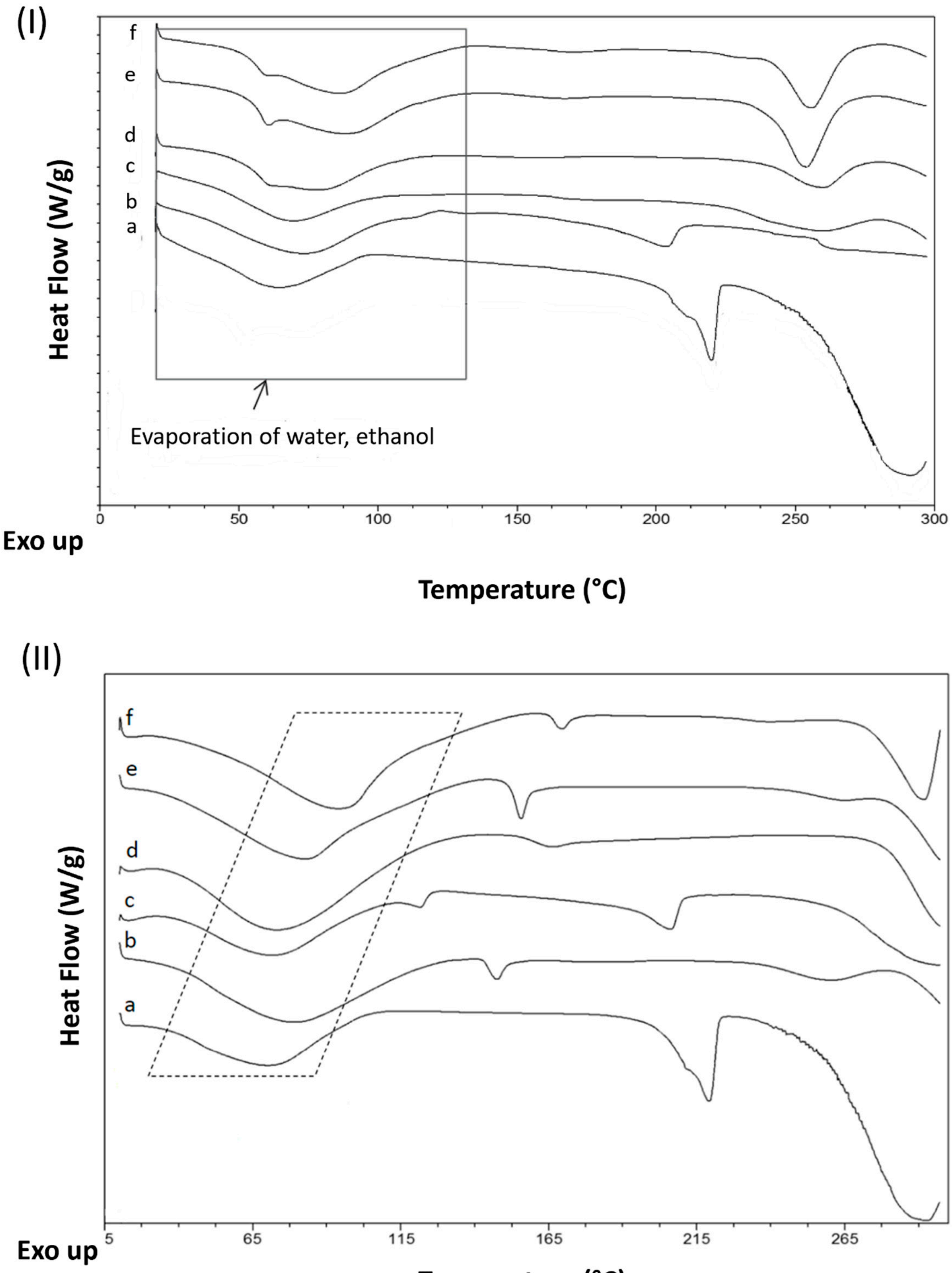

Figure S5. DSC thermogram of (I) non-crosslinked and crosslinked (II) hybrids scaffolds. (a) 5%, (b) 10 %, (c) 15 %, (d) 20%, (e) 25%, and (f) 30%. All the peaks describe an endothermic transition.

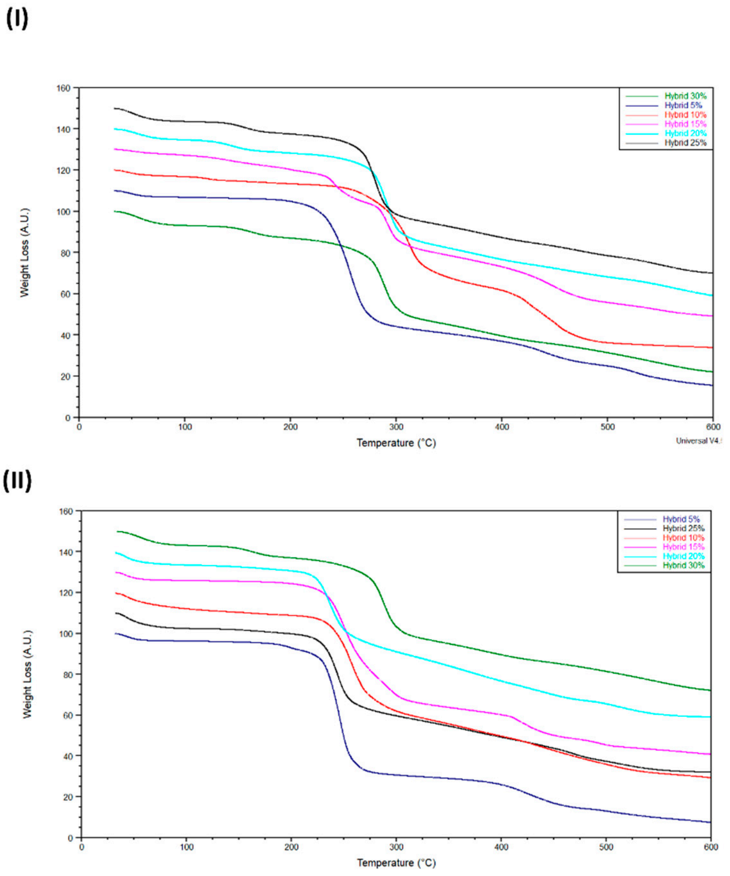

Figure S6. TGA thermograms of PVA/ Bg hybrid scaffolds. (I) before crosslinking and (II) after crosslinking reaction.

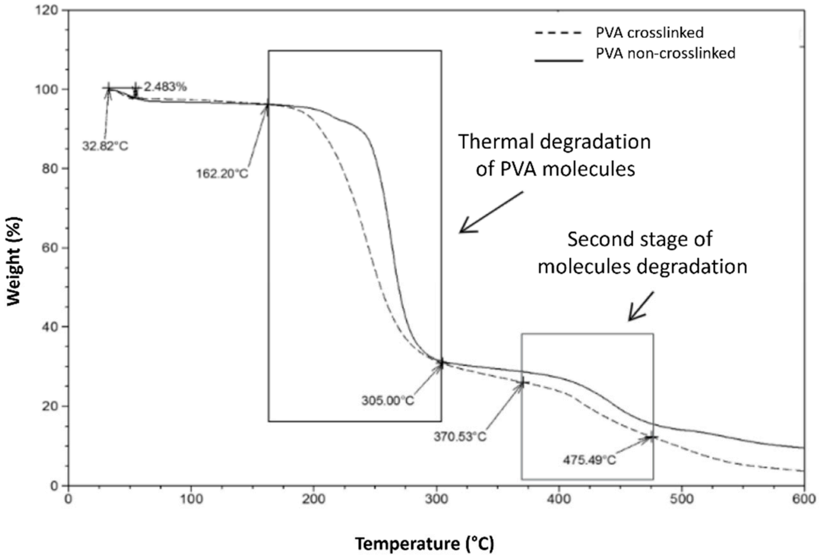

Figure S7. PVA thermograms before and after crosslinking.

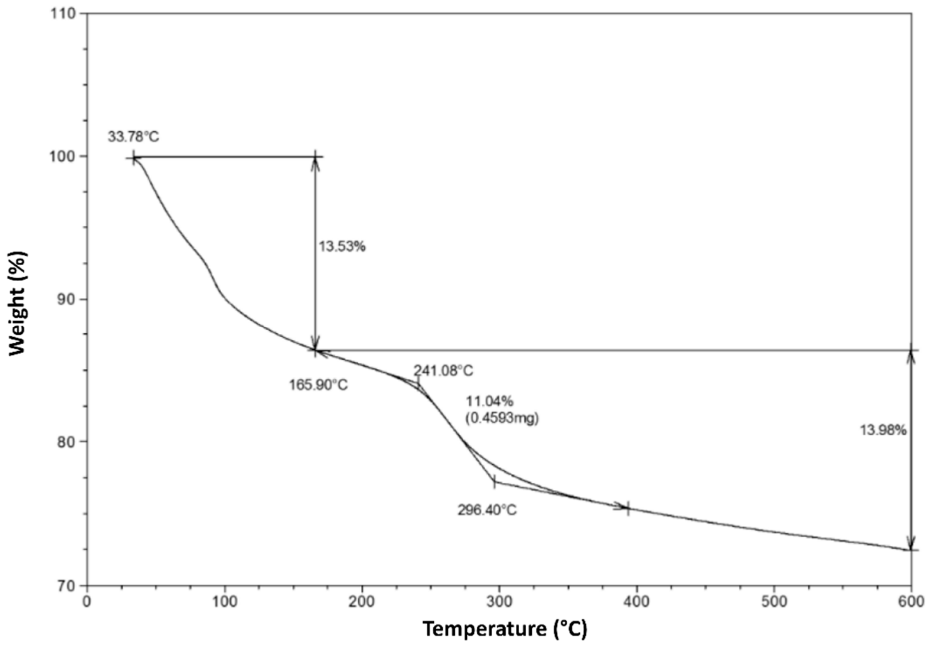

Figure S8. Bioglass TGA thermogram.

Determination of standard deviation in fiber diameter measurement in electrospun scaffolds.

The procedure for calculating the uncertainty associated the fiber diameter ($\bar{X}$) is explained below. Fiber diameters were measured in microns on 30 different fibers under repeatability conditions. For example, the average diameter was 0.127 µm for PVA fibers before chemical crosslinking. The non-statistical uncertainty was determined by considering the uncertainty due to the resolution provided by the ImageJ program: u_B (x) = 0.001 µm. To determine the statistical uncertainty, it is necessary to know the standard deviation of the sample.

$$s(x)=\sqrt{\frac{\sum_{i=1}^{30}(x_i-\bar{x})^2}{n-1}} = 0.009 \ \mu m, \quad (1)$$

The uncertainty type A is obtained: (u_A (x))

$$u_A(x)= s(x)/\sqrt{n} = 0.002 \ \mu m, \quad (2)$$

Finally, the combined uncertainty, u_C (x), was calculated, for which we substitute the values of u_A (x) and u_B (x).

$$u_C(x)= \sqrt{u_A^2(x) + u_B^2(x)} = 0.002 \ \mu m, \quad (3)$$

The value of the diameter of the PVA fibers for test a) with the associated uncertainty is therefore: (0.127± 0.002) µm.
